# Psychotic Symptoms in Heroin Withdrawal: A Case Report

**DOI:** 10.7759/cureus.12620

**Published:** 2021-01-11

**Authors:** Surabhi Sharma, Prerak Kumar, Romil Singh, Guneet S Sidhu, Kaushal Shah

**Affiliations:** 1 Psychiatry and Behavioral Sciences, Lady Hardinge Medical College & Smt. S.K. Hospital, New Delhi, IND; 2 Department of Critical Care Medicine, Mayo Clinic, Rochester, USA; 3 Department of Gastroenterology and Hepatology, Mayo Clinic, Rochester, USA; 4 Psychiatry, Griffin Memorial Hospital, Norman, USA

**Keywords:** opioid withdrawal, psychosis, heroin, fluctuating course, opioid use

## Abstract

Opiate withdrawal-induced severe exacerbation of psychosis after the sudden withdrawal of an opiate is a known yet uncommon clinical manifestation. We present the case of opiate withdrawal-induced psychosis in a 25-year-old married male patient without any prior psychiatric history of illness, family history, or past hospitalization records. The patient presented with psychotic symptoms such as irritability, delusions of parasitosis, and auditory and visual hallucinations at the time of hospitalization. Symptoms were in a fluctuating course and were not constant throughout the day. Through this case report, our aim is to present a rare instance of heroin-withdrawal-induced psychosis and its successful treatment with antipsychotics.

## Introduction

Illicit compounds, such as heroin, synthetic opioids, such as fentanyl, and prescription legal analgesics, such as codeine, oxycodone, hydrocodone, and morphine, fall under the class of drugs known as opioids [1]. When used by a patient under a health care provider’s direction, prescription opioid analgesics help alleviate and control pain. However, misusing any opioid, including prescription opioids, can result in dependence and addiction [1,2].

The psychological and physical symptoms that occur after abrupt cessation or dose reduction of opioids, also known as opioid withdrawal, are very unpleasant and distressing. Acute typical opioid-withdrawal symptoms are rhinorrhoea, lacrimation, generalized pain, nausea, and yawning that may present after abrupt discontinuation of substance within three to five days of regular opioid use [1]. Generally, psychotic symptoms are rare with opioid withdrawal, but few studies have reported their associations with withdrawal of synthetic opioids such as tramadol, oxycodone, buprenorphine [2,3]. Opiate agonists have stimulating properties on mu receptors in the brain to modify dopamine flows, and their release interferes with postsynaptic dopamine action. Endogenous opioid endorphins are inhibitory neuromodulators of dopamine activity. Absence or deficiency of endorphins may increase dopamine release and turnover and result in psychosis [4].

According to literature, tramadol withdrawal may present with atypical symptoms of psychosis, such as irritability, when a patient abruptly stops usage with a dose of 300 mg per day. Although opioid-induced psychoses are well documented in the literature, there might be an antipsychotic effect in some opioids because of action at dopamine and mu receptors [5]. However, withdrawal deficiency leading to psychosis is still rare and uncommon in clinical scenarios.

## Case presentation

A 25-year-old married male came to the outpatient psychiatry clinic with his mother as an informant with a total duration of heroin use of about two years. He complained of runny nose, diarrhea, headache, and cramping pain in his legs in outpatient settings. The clinical opiate withdrawal scale (COWS) score was 13, indicating moderate opioid withdrawal. Interestingly, he also complained of insects crawling over his chest. On further questioning, he described insect color and size. He also reported that he kept hearing the voice of a female who lived in his neighborhood instructing him to do things, but his mother denied such instances and said no such female lived in their neighborhood. The patient was admitted to the psychiatry inpatient department. During his inpatient stay, care was provided according to the hospital protocol. In addition to three meals in the form of breakfast, lunch, and dinner, the patient was provided with snacks between the meals. Family visitation was granted during the day but they were not allowed to stay in the hospital. The patient and his family members complied with hospital policies and staff. Relationship between patients and family members was cordial before and during inpatient hospitalization. His blood investigations were within normal limits, but his electrolyte report showed hyponatremia with a serum sodium level of 131 mEq/L (normal range: 135-145 mEq/L). The patient was admitted and started on buprenorphine 4 mg tablets in divided doses, diclofenac 100 mg tablet on a pro re nata basis, and olanzapine 2.5 mg tablet at bedtime in the night. After two days in the ward, his COWS score reduced to four. At this time, he only complained of anxiety and yawning symptoms. However, he reported a continuance of psychotic symptoms. He reported that a female voice still ordered him to run from the ward and do some rituals for her. He also said that insects are all over his hair and chest. The patient also described the female wearing a blue saree on further exploration, which he saw mainly during afternoons only for a few minutes.

Due to persistent hallucinations and delusions, olanzapine dose was increased to 10 mg. Within the next three days, visual hallucinations were not reported, but the patient reported that insects were still continuously troubling him by causing itching all over his body. Therefore, computed tomography scan of the head was scheduled to rule out the organic cause of psychotic symptoms, but the patient’s family denied consent due to financial constraints and low economic status. After stabilizing the patient, he was discharged on buprenorphine 2 mg tablet, olanzapine 12.5 mg tablet, and clonazepam 1 mg tablet once daily for ten days. After ten days of follow-up, the patient reported delusional parasitosis, with very few fragmentary auditory hallucinations. The patient’s mother reported excessive eating, sedation, and mild dizziness with no withdrawal symptoms; COWS score was zero. At this visit, olanzapine was discontinued, and the patient was started on 2 mg risperidone twice daily, and a two-week follow-up was advised. After two weeks, the patient reported about 50% improvement in the delusion of parasitosis. Risperidone dose was increased to 3 mg twice daily, with buprenorphine 2 mg once daily and clonazepam 0.75 mg once daily. A follow-up visit was scheduled after two weeks. The patient didn’t report for further follow-up, but the mother came to the outpatient clinic for his medications. According to the mother, the patient showed about 75% improvement on risperidone and complied with medications. The unique clinical finding of psychotic symptoms after opioid withdrawal is uncommon in literature and clinical practice compared to other substances. This case provides more clarity in terms of dual diagnosis in our routine practices.

## Discussion

Although the occurrence of psychosis after a sudden withdrawal of opioids is uncommon as part of the opioid withdrawal syndrome, a few cases of psychotic symptoms have been described in the literature [6,7]. A study presented the case of a 57-year-old male with an intrathecal morphine pump for his chronic back pain for five to six years. The patient suddenly started to show psychotic symptoms, mainly auditory, visual, and olfactory hallucinations with disorganized behavior, paranoid ideas, and persecutory delusions after abrupt tramadol withdrawal [8].

Another study by Senay et al., consisting of 422 patients who were prescribed tramadol for managing opioid abuse or dependence, reported atypical and typical opioid withdrawal symptoms. Atypical opioid withdrawal symptoms include hallucinations, suspiciousness, panic attacks, and delirium. Typical opioid withdrawal symptoms consist of anxiety, diarrhea, insomnia, lacrimation, rhinorrhea, and sweating [9]. Maremmani et al. found a relation between multiple drug abuse and psychosis, but the association is unclear in case of opioids [10]. Weibel et al. and Karila et al. reported clinical cases with abrupt psychiatric onset symptoms within a few days of buprenorphine withdrawal. Symptoms presented as aggression, suicidal thoughts, and hearing voices within a few days post sudden withdrawal of buprenorphine in the dose range of 6 to 8 mg per day [6,7].

## Conclusions

This case highlights the importance of early use of antipsychotics in rare cases of opioid withdrawal accompanied by psychotic symptoms. It is essential to conduct a thorough psychiatric evaluation of patients presenting with psychotic symptoms to rule out their precipitating causes, including other comorbidities and family psychiatric history. It will mainly help avoid the emergence of distressing acute psychotic symptoms in rare associations with substances such as opioids, as in this case. The pathophysiology of opioid-induced psychosis and their antipsychotic mechanism warrants further investigation.
